# Improving Chitosan Hydrogels Printability: A Comprehensive Study on Printing Scaffolds for Customized Drug Delivery

**DOI:** 10.3390/ijms24020973

**Published:** 2023-01-04

**Authors:** Sara Cardoso, Francisco Narciso, Nuno Monge, Ana Bettencourt, Isabel A. C. Ribeiro

**Affiliations:** 1 Research Institute for Medicines (iMed.ULisboa), Faculty of Pharmacy, Universidade de Lisboa, Avenida Prof. Gama Pinto, 1649-003 Lisboa, Portugal; 2 Faculdade de Ciências e Tecnologia, Universidade Nova de Lisboa, Campus de Caparica, 2829-516 Caparica, Portugal; 3 Centro Interdisciplinar de Estudos Educacionais (CIED), Escola Superior de Educação de Lisboa, Instituto Politécnico de Lisboa, Campus de Benfica do IPL, 1549-003 Lisboa, Portugal

**Keywords:** chitosan, scaffolds, starch, 3D printing, drug-delivery

## Abstract

Chitosan is an interesting polymer to produce hydrogels suitable for the 3D printing of customized drug delivery systems. This study aimed at the achievement of chitosan-based scaffolds suitable for the incorporation of active components in the matrix or loaded into the pores. Several scaffolds were printed using different chitosan-based hydrogels. To understand which parameters would have a greater impact on printability, an optimization study was conducted. The scaffolds with the highest printability were obtained with a chitosan hydrogel at 2.5 wt%, a flow speed of 0.15 mm/s and a layer height of 0.41 mm. To improve the chitosan hydrogel printability, starch was added, and a design of experiments with three factors and two responses was carried out to find out the optimal starch supplementation. It was possible to conclude that the addition of starch (13 wt%) to the chitosan hydrogel improved the structural characteristics of the chitosan-based scaffolds. These scaffolds showed potential to be tested in the future as drug-delivery systems.

## 1. Introduction

In the last few years, 3D printing technology has shown great application in many areas, including in healthcare. This technology can be used to create medicines and medical devices including diagnostic tests or a functional organ with certain characteristics which is not possible to be created with traditional methods [1,2,3]. In addition, in a healthcare setting it is possible to produce devices or medicines, with unique features considering the individual characteristics of each patient. It can also allow a reduction in time and cost production, which can be decisive in the context of the health field [4].

Depending on the final application different, 3D structures can be produced including scaffolds. Scaffolds are porous structures that can have different geometries. Scaffolds act like artificial extracellular matrices supporting three-dimensional tissue formation [5]. Moreover, these structures can also act as a delivery system of active compounds included in the matrix or loaded into the pores [6]. This feature creates a highly dynamic structure, since it is possible to produce fixed matrices that allow the incorporation of components considering a given prescription.

Although the 3D printing technology shows many advantages, there are also some limitations that need to be considered. For example, depending on the 3D printing method and application field, the type of suitable materials will vary, and in some cases, the availability in materials is short. In addition, the printing process will depend on some critical characteristics that are related to the final application, the desired structure, type of material and cost. Typically, the most common materials used in a healthcare setting are polymeric and those are mostly used as a filament ready to be extruded by thermal means. Another possibility is the use of hydrogels suitable for syringe extrusion [7]. When it comes to syringe extrusion-based 3D printing, hydrogels are one of the best materials to use as the main component of the bio-inks [8,9,10]. This type of 3D printing technique requires a bio-ink with a suitable viscosity and hydrogels can fit this purpose. Furthermore, the bio-inks need to be characteristically shear, thinning to compensate for the high shear stress to which the material is subjected to during its extrusion in the 3D printing process [9,10].

Among different polymers suitable for syringe extrusion. chitosan is often selected [11]. Chitosan is one of the most used polymers due to its high biocompatibility with different materials and cells, non-toxicity and antimicrobial activity, that can be applied in different areas, including biomedical engineering, clinical medicine, pharmaceuticals, the food industry and bioremediation [11,12,13,14,15,16,17,18]. It is a natural linear polymer composed by N-acetyl-2-amino-2-deoxy-d-glucopyranose units (acetylated units) linked by β-(1→4)-glycosidic bonds to 2-amino-2-deoxyd-glucopyranose units (deacetylated units) [19].

Chitosan is a natural linear polymer that can be produced through partial deacetylation of chitin, as a result of hydrolysis in an extreme alkaline environment with high temperatures [12,20,21,22,23]. In nature, chitin can be found in the constitution of a variety of different living forms, mostly on the shells of sea creatures such as crustaceans and shrimps [23,24], but also from the exoskeletons of insects [22,24] and fungi [24]. Its biocompatibility allows it to be used in the production of drugs and medical devices, such as tablets, patches, implants and wound dressings [20,24]. In addition, chitosan presents a high biodegradability [12,20,21,22,23,24], allowing the production of modified release drugs [20,24], which combined with its antimicrobial activity, have an effect in the healing process and are suitable for wound dressings [20,21,22,24]. Moreover, chitosan-based hydrogels may present suitable rheological properties making this material relevant for 3D printing. However, the use of pure chitosan faces many challenges due to the presence of poor mechanical properties, which can create inadequate structures, with poorly defined pores, that will result in a poor printability [21,25]. Several authors suggest that one solution to enhance the printability of chitosan-based scaffolds could be achieved by the addition of other materials [21,26], such as starch [25], alginate [27], pectin [28] and collagen [29]. Starch is a polymer that has been highlighted due to its rheological properties, presenting shear-thinning behavior, essential characteristics for 3D printing [21,30]. In this context, the aim of this study was to achieve a chitosan-based model formulation suitable for the 3D printing of a high-quality net structure intended to be used in customized therapeutics. To produce chitosan hydrogels suitable for a 3D extrusion-based method, an optimization study was carried out. The study aimed to understand which parameters influenced the printability of the final structure. In the end, after selecting the optimal parameters, a design of experiments was conducted in order to study the influence of starch supplementation to the chitosan hydrogel and produce scaffolds with properties suitable for customized drug delivery systems.

## 2. Results and Discussion

### 2.1. Pure-Chitosan Hydrogels Evaluation

Two different hydrogels with a chitosan composition at 2.0 wt% and 2.5 wt% were first produced in order to carry out an optimization study to select which parameters influenced the most in the printing of the chitosan scaffolds. Preliminary studies (data not shown) showed that chitosan concentrations of 1.0 wt% and 3.0 wt% were not suitable for printing since they were too fluid or too viscous, respectively. Therefore, in-between concentrations were chosen.

**Figure 1:**
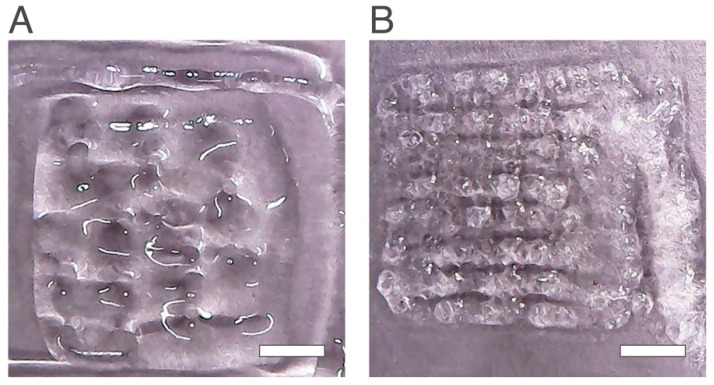
Scaffolds obtained using the same printing conditions and different chitosan percentages. (**A**) Chi 2.0 wt% and (**B**) Chi 2.5 wt%. The scale bar measures 5 mm.

When fixing the printing conditions at a printing speed of 20 mm/s, glass bed temperature at room temperature, deposit speed of 7 mm/s, compensate at 0 µL, purge at 0 µL infill speed of 5 mm/s, flow speed at 0.25 mm/s and layer height at 0.25 mm, different scaffolds were obtained with 2.0 wt% (Chi 2%) and 2.5 wt% (Chi 2.5%) chitosan hydrogels (Figure 1).

Under the same printing conditions, the scaffold Chi 2.5% (Figure 1B) showed a better structure than Chi 2.0% (Figure 1A). It was also possible to identify several pores and the matrix of the scaffold contrary to Chi 2% where the printing failed, and a poor structure was produced with only some pores with poorly defined boundaries. Several replicas were performed and 2.5 wt% chitosan showed higher printability properties. Previous studies also showed that the concentrations of chitosan used to produce scaffolds have influence in the printability; the higher chitosan concentrations produce structures with higher quality [31]. Thus, the hydrogel selected for further studies was the Chi 2.5%.

After selecting the hydrogel that had the best printability properties regardless of the printing conditions applied, several printing parameters were studied to understand which ones would impact on chitosan scaffolds. For the same printing conditions, the printing parameters that showed the highest impact in the final printability were the nozzle diameter, flow speed and layer height.

Regarding the nozzle diameter, two different tips were used, one with a larger diameter (0.58 mm) and another with a smaller one (0.41 mm). Regarding the nozzle tip diameter, when the tip with the smallest diameter was used the printing process failed because only a small portion of the hydrogel was extruded from the nozzle. On the other hand, when the tip with the larger diameter was used, the hydrogel was extruded through the tip and it was possible to form a 3D structure on the glass bed. In an extrusion-based 3D process, one of the major problems related to the method, is the possibility of tip clogging [32]. This was observed with the tip with a diameter of 0.41 mm thus showing inadequate diameter for printing the chitosan hydrogel. Based on these results, the nozzle tip with 0.58 mm was selected for further use.

**Figure 2:**
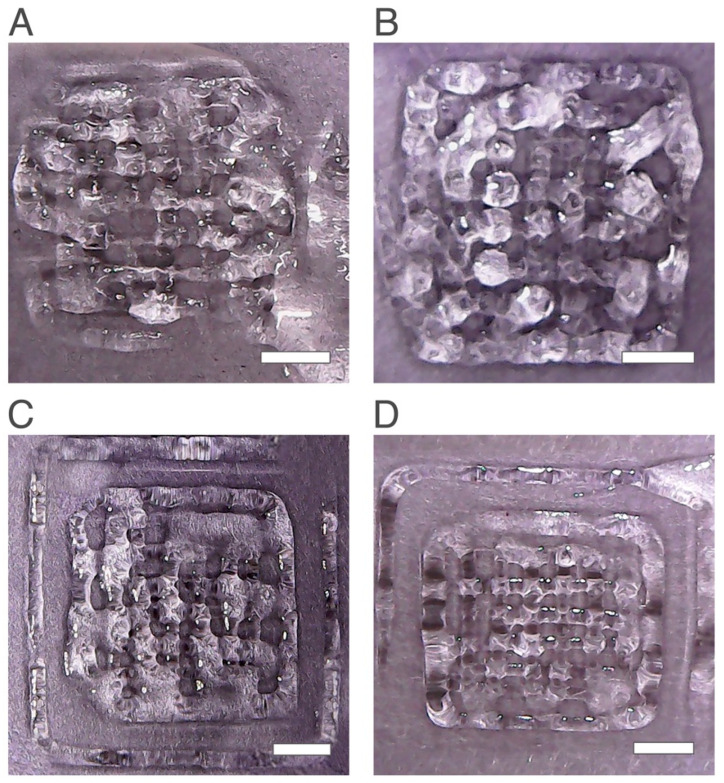
Printed scaffolds with 2.5 wt% chitosan obtained with the same printing conditions except layer height (LH) and flow speed (FS). (**A**) 0.25 mm LH and 0.5 mm/s FS, (**B**) 0.41 mm LH and 0.50 mm/s FS, (**C**) 0.41 mm LH and 0.25 mm/s FS and (**D**) 0.41 mm LH and 0.15 mm/s FS. The scale bars measure 5 mm.

Regarding the layer height parameter, two different heights were studied: 0.15 mm and 0.41 mm. As shown in Figure 2A,B, the scaffold printed with a layer height of 0.41 mm showed a better structure with a more defined matrix when compared to the scaffold with a smaller layer height. Previous studies reported that changing the layer height has a direct impact on the printability of the final scaffold [33]. Moreover, a relationship between the increase in layer height with printability has been previously established [33]. Higher layer height values will produce a better structure with higher printability and accuracy and lower values will result in lower resolution and quality, being in agreement with the obtained results [33].

When fixing the layer height at 0.41 mm and only changing the flow speed parameter, changes in the obtained scaffolds were observed (Figure 2B–D). When the flow speed value decreased from 0.5 mm/s to 0.25 mm/s and 0.15 mm/s, the quality of structures improved by showing a higher number of pores and a matrix with higher resolution. These results agreed with previous studies stating that flow speed has an impact on printability and fidelity of the printed scaffold [34,35,36]. Hence, for this study the printability increased with the decrease in the flow speed, until an optimal value of 0.15 mm/s was reached.

Despite being possible to print scaffolds with the chitosan hydrogel, the structures obtained did not match the desired quality. One of the major limitations when using only chitosan to print 3D structures is the lack of mechanical properties that have been reported in previous studies [25,28,37,38]. Thus, to enhance the final printability and improve the mechanical properties of chitosan, some authors suggest the addition of other materials to the chitosan [21], such as starch [25]. In order to understand if other materials would improve the printability of chitosan, new hydrogels were produced by combining starch with 2.5 wt% chitosan. Previous studies indicate that the addition of starch to a chitosan-based hydrogel may increase the printability of the final scaffold [25,30].

### 2.2. Design of Experiments

From the optimization study carried out (2.1.), it was concluded that it is possible to use pure chitosan hydrogels to produce 3D objects. However, its use has shown some limitations regarding its final structure. To improve chitosan printability, a common strategy is to include other polymers in the bio-ink formulation, namely, gelatin, starch, collagen, pectin and alginate [25,26,27,28,29]. In this work, starch was chosen for being a common pharmaceutical excipient, affordable and easy to obtain.

Currently, there is lack of information regarding what will be the ideal concentration of starch that should be added to chitosan. To reach the optimal starch %, many experiments would have to be performed which could be time and cost consuming. Therefore, to ensure that the selected experiments produce the maximum amount of relevant information, a design of experiments (DoE) tool can be used instead of an empirical approach of “trial and error”. Specifically, a full-factorial design was used allowing to study the influence of experimental factors, to understand which factors have an impact on the study and which factors can be discarded [39,40]. Moreover, a full-factorial design allows to study the variation among different factors, from the minimum to maximum value that can be used, and understand their effects and interactions between the factors in a study [39,40].

Thus, a design tool was used to minimize the number of experiments performed when studying a wide range of concentrations. From the optimization study carried out, the flow speed and layer height were the two parameters which had the greatest impact on the quality of the printed scaffolds. Thus, a design of experiments was conducted by studying the influence of starch amount supplementation in relation to the variation in the layer height and flow speed.

Table 1 presents the results for the total number of pores (*NP*) and printability (*Pr*). Regarding the *NP* response, from the 11 runs, the experiment number 1 (34 *NP*) and 10 (32 *NP*) showed the highest *NP* value. Although these two runs demonstrated the highest *NP* of all experiments, this value was far away from the desired theoretical value of 49. On the other hand, the experiment number 7 and 10 showed the highest printability values, 0.94 and 0.93, respectively. In theory the scaffold that presents the highest printability and/or number of pores would be the one that presents the better quality, fidelity, structure and resolution.

**Figure 3:**
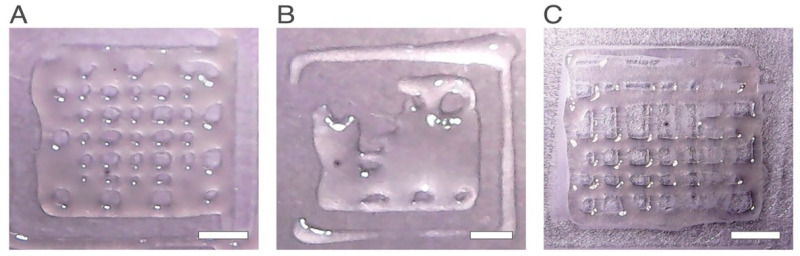
Printed scaffolds, for the experiments with the highest printability and number of pores. (**A**) Experiment number 1. High printability and high number of pores (**B**) Experiment number 7. Although the formed pores presented a relatively high printability score, very few pores were formed. (**C**) Experiment number 10. High printability and high number of pores result. The scale bars measure 5 mm.

However, a qualitative analysis of the images of the scaffolds (Figure 3) obtained for the experiments with the highest values of response (*NP* or *Pr*) shows that in experiment number 7, a poor quality scaffold with only some formed pores was produced (Figure 3B).

The number of pores and printability can be used to quantify the performance of the printing process for each experiment obtained; however, by using these responses separately, a misinterpretation of results may occur. For example, if the theoretical total pore number is 49, and one scaffold presents only 1 pore, this scaffold can present a printability of 1 if it presents a high resolution with well-defined boundaries. On the other hand, a scaffold can present a total of 48 pores out of 49, and have a printability inferior to 1 if the pores produced are not equal to each other and have poor resolution.

To evaluate the combined effect of the two responses, the total number of pores and the printability, a new formula was here presented, and the combined variable was designated as “Qscaffold” for “Quality-scaffold”.

**Figure 4:**
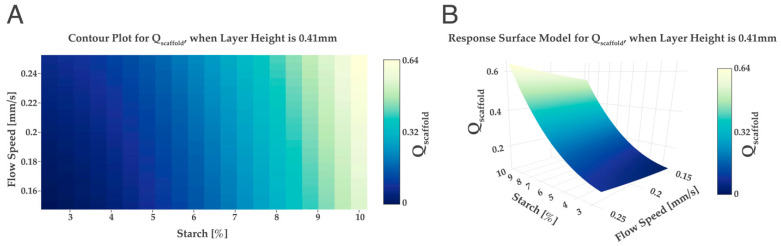
Qscaffold obtained for different wt% values of starch and flow speed when the layer height is kept at a constant value of 0.41 mm. Contour plot (**A**) and response surface model (**B**).

According to the results presented in Table 2, the experiment number 10 and 1 showed the highest Qscaffold score was associated with the scaffolds that presented the highest quality, i.e., higher pore number obtained combined with a well-defined matrix. In the experiment number 1, a flow speed of 0.25 mm/s, a layer height of 0.41 mm and a starch % of 10 were used. In experiment 10, a flow speed of 0.15 mm/s, a layer height of 0.41 mm and a starch % of 10 were used. Moreover, the scaffolds in experiment number 10 and 1 showed two factors in common, the starch percentage and the layer height. Regarding the parameter flow speeds and layer heights, the results for the design of the experiments were in accordance with the results obtained for the Chi 2.5 wt%, where the optimal values for both parameters were 0.15 mm/s and 0.41, respectively. Hence, to study the influence of different starch percentages a new model was applied by fixing the layer height at 0.41 mm and obtaining a surface and a contour plot (Figure 4).

In Figure 4, it was possible to observe a relation between Qscaffold and the percentage of starch, the greater the percentage of starch, the greater the Qscaffold value obtained. Previous studies found out that the addition of starch will improve the scaffold’s printability; the higher the percentage of starch, the better the quality of the printed scaffold [25,41]. Thus, for the present design of experiments, the ideal starch percentage was set at 10 wt% starch.

### 2.3. Simulations and Experiments with 13 wt% of Starch

**Figure 5:**
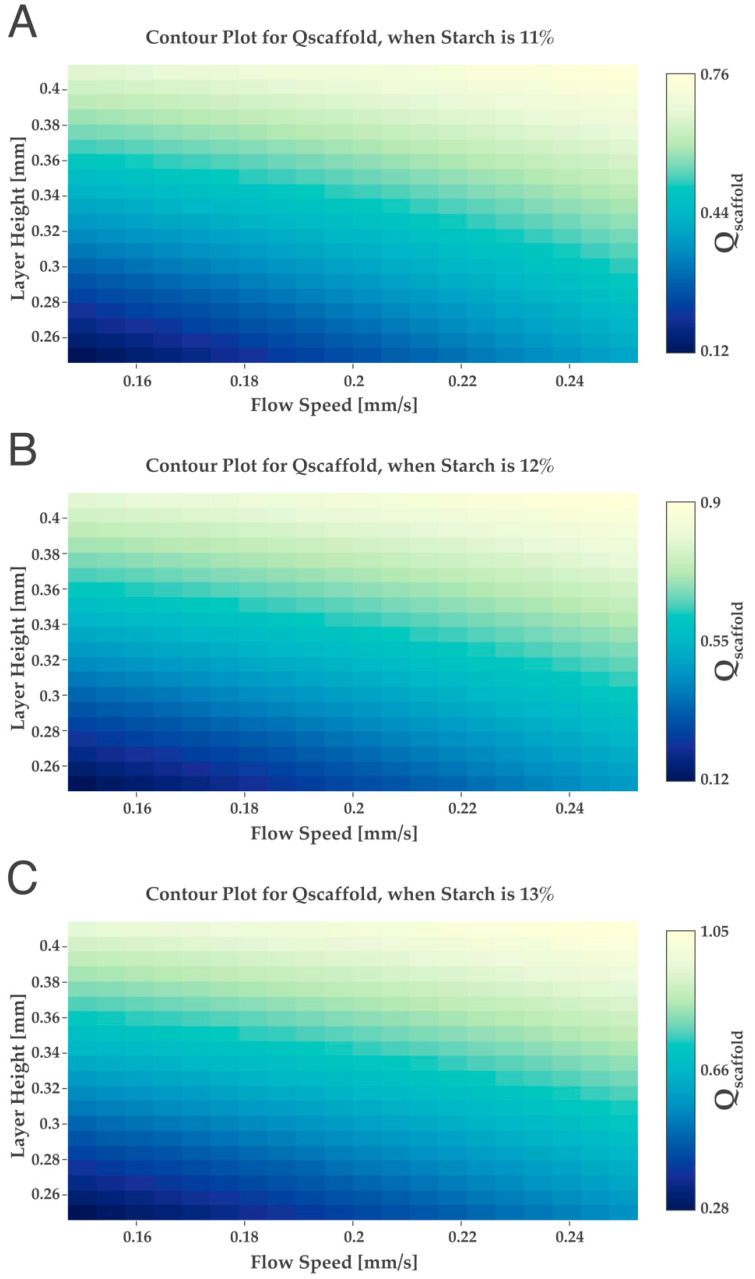
Simulations using different starch percentages (**A**) 11 wt%, (**B**) 12 wt% and (**C**) 13 wt%.

The maximum starch concentration used in the design of experiments was 10 wt%; however, the maximum Qscaffold obtained was 0.61, which was quite far from the ideal (Qscaffold value of 1). This could mean that to achieve a higher Qscaffold, it is necessary to use hydrogels with a higher percentage of starch. To study if the raising of the starch concentration would increase the Qscaffold, several simulations were conducted using higher starch values (>10 wt%) different from the design of experiments, until a Qscaffold of 1 was achieved, using the “desice.on software online”. For this, the starch variable was established as a constant factor, and the Qscaffold obtained for the selected starch % was obtained in a function of the variation in the layer height (0.25 to 0.41 mm) and flow speed (0.15 mm/s to 0.25 mm/s). Therefore, three different percentages of starch were fixed (11, 12 and 13 wt%) in a function of the variation in the flow speed and layer height, and the respective contour plots were obtained (Figure 5).

Regarding the results shown in Figure 5, when selecting a starch wt% of 11 and 12, the maximum Qscaffold obtained was lower than 1. However, when selecting 13 wt%, it was possible to obtain a Qscaffold of 1. If we keep increasing the starch wt% to values higher than 13, a Qscaffold of 1 was possible to be obtained until 18 wt%. Experimental studies by others show that the increase in starch amount in hydrogels will produce stronger structures, homogeneous with more pores and with well-defined boundaries, complying with the simulations performed in our study [42]. Thus, after obtaining, by simulation, the minimum theoretical value of starch to reach a Qscaffold of 1, novel hydrogels were produced with 13 wt% of starch.

**Figure 6:**
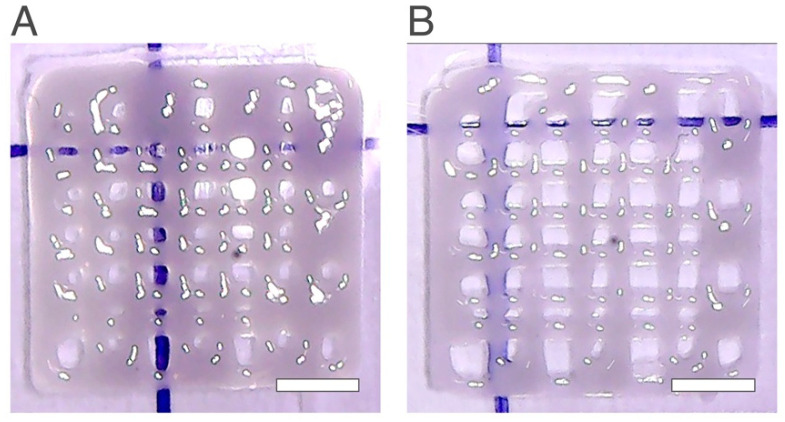
Printed scaffolds with 2.5 wt% chitosan and 13 wt% starch with different flow speed values (**A**) 0.25 mm/s (**B**) 0.15 mm/s. The scale bars measure 5 mm.

Table 3 shows the results obtained for the hydrogels with the new starch percentage. According to these results, the Qscaffold obtained with the flow speed at 0.15 mm/s was close to 1. Figure 6 shows the printed scaffolds obtained with 13 wt% of starch.

When comparing the scaffolds with 13 wt% with the scaffolds obtained in the design experiment number 10 (Figure 6), the number of pores increased from 32 to 40 and 46, the pore resolution increased, and the final net became more defined. In addition, for the scaffolds with 13 wt% starch, there is another consideration that can be pointed out regarding the flow speed value. For the design of experiments, the experiment that had the highest Qscaffold was the one that presented the highest quality of the final structure, and to print this scaffold, it was necessary to use a flow speed value of 0.15 mm/s and a layer height of 0.41 mm.

When studying the new wt% of starch, two different flow speed values were used, 0.15 mm/s and 0.25 mm/s, the same as those used in the design of experiments. Regarding the flow speed value, when using a higher value, the number of pores decreased and the Qscaffold value was inferior to when using a lower flow speed value (0.15 mm/s). This new consideration is quite important since the only printing condition change for both scaffolds presented in Figure 6A,B was the flow speed value. A decrease in flow speed showed a structure with higher quality with a higher number of pores, as in agreement with the results obtained previously.

For the same printing conditions, the scaffolds composed by chitosan 2.5 wt% and starch 13 wt% were demonstrated to have a better appearance, better structure, the layers adhered better between them, the printability was the one that was closest to 1 (*Pr* = 0.99) and the total number of pores formed was the one that came closest to the desired theoretical value (49 pores). Thus, the optimal starch amount was 13 wt%. It can be concluded that the addition of starch in a chitosan hydrogel improves the final structure enhancing the printability of the hydrogel.

Regarding the results for the starch at 13 wt%, increasing the amount of this polymer in a chitosan hydrogel at constant concentration (2.5 wt%) led to an increase in quality, fidelity and resolution of the scaffolds appearance. As indicated in previous investigations, the incorporation of higher concentrations of starch would result in better properties in terms of printability, which corroborates with the results obtained in the DoE and when simulating higher starch wt% [25,41].

Recent investigations have shown that the addition of other polymers to chitosan has resulted in better results in terms of printability [38], more specifically related to the combination of starch [25]. However, these investigations lack information associated with the optimal values and printing conditions necessary to produce the chitosan–starch hydrogels and the scaffolds. In a study performed by Butler et al. in which chitosan–starch scaffolds were studied to be used as tools for neural applications, it was shown that the production of chitosan–starch scaffolds at a 50%:50% ratio presented a promising formulation to be used for neural cells [25]. Our work presents a comprehensive study of the chitosan and chitosan–starch scaffolds, given information about how to produce and print chitosan hydrogels, and how to use these hydrogels combined with starch. On the other hand, the production of the chitosan solution and starch solution, before being combined for the present work, only required the incorporation of two reagents, acetic acid and water, respectively. This simple formulation allowed the formation of scaffolds with suitable properties for drug delivery, and at the same time, reducing unnecessary costs and material waste. Moreover, the matrix produced can be used to incorporate a desired active component in the matrix or loaded directly into the pores. On other hand, knowing the printing conditions to create the best structures, allows it to be customized not only in terms of incorporating new components, but also changing its geometric properties (size, height, etc.). Changing the geometric properties allows the changing in the configuration of the scaffold by taking into account the place of application, which can be very important in the production of delivery systems for wound dressings.

### 2.4. Viscosity Evaluation

Viscosity is an important parameter that needs to be considered when selecting the materials to produce the hydrogels. Since the material has to pass through the nozzle tip, it is necessary that it has an adequate viscosity behavior as a function of shear stress [43]. The 3D printing process chosen for the present study, an extrusion-based method, and according to previous studies, shows that it is mandatory that the materials used for this type of process present a shear-thinning behavior for the hydrogel be able to extrude through the nozzle tip [44,45,46,47].

**Figure 7:**
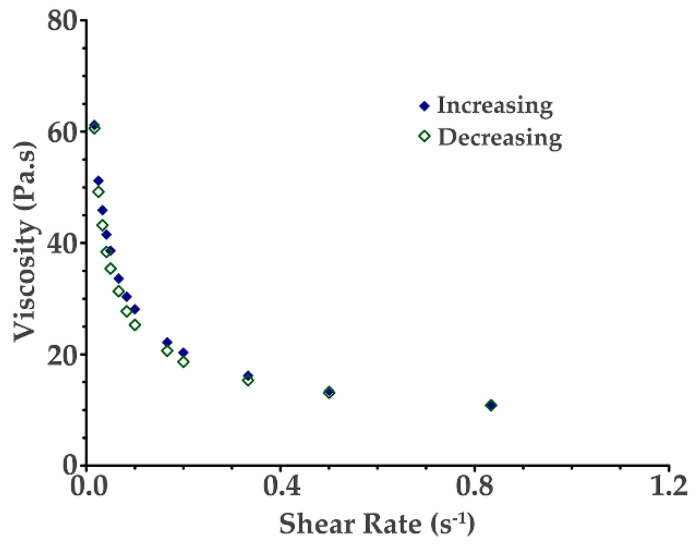
Viscosity as a function of shear rate for the chitosan–starch (2.5 wt% chitosan and 13 wt% starch) hydrogel.

Figure 7 shows the viscosity vs. shear rate for the chitosan–starch (2.5 wt% chitosan and 13 wt% starch) hydrogel. The hydrogel produced can be classified as a non-Newtonian fluid with a shear-thinning behavior, that is the decrease in the viscosity with the increase in the shear rate.

Moreover, the viscosity of the chosen materials should be low enough for the hydrogel be extruded from the nozzle tip and high enough for the printing layers to adhere to each other, maintain the desired shape, and print strong and adequate structures that are stable over time [48,49]. If the viscosity of the hydrogel is too high, the hydrogel will not be able to pass through the nozzle tip and problems related to clogging will happen [7,50]. According to the results obtained, the chitosan–starch hydrogel was demonstrated to have the proper viscosity for 3D printing.

## 3. Materials and Methods

### 3.1. Chemicals and Reagents

The following chemicals were used: chitosan (medium molecular weight; deacetylation degree 75–85%) from Sigma Aldrich (Steinheim, Germany); starch from Merck (Darmstadt, Germany); glacial acetic acid from Scharlau (Barcelona, Spain) and water purified by a Milli-Q purification system (Millipore).

### 3.2. Production of Chitosan Hydrogels

Several hydrogels were prepared using pure chitosan and chitosan combined with starch. To prepare the pure chitosan hydrogel, a proper amount of chitosan was weighed and dissolved in 1 wt% aqueous acetic acid solution in a glass beaker. The final solution was transferred to a magnetic stirrer and left to stir for 4 h at 800 rpm. In addition, to prepare the starch solution, the proper amount of starch was weighted, added of milli-Q water in a glass beaker and stirred for 4 h at 800 rpm and 95 °C.

To produce the hydrogel composed of chitosan and starch, the appropriate volume of each solution was combined and stirred at 800 rpm and 80 °C for 3 h. The final solutions were composed of pure chitosan at 2 and 2.5 wt%, chitosan–starch with chitosan 2.5 wt% and different starch percentages (2.5, 6.3, 10 and 13 wt%).

### 3.3. Bioprinter Setup

**Figure 8:**
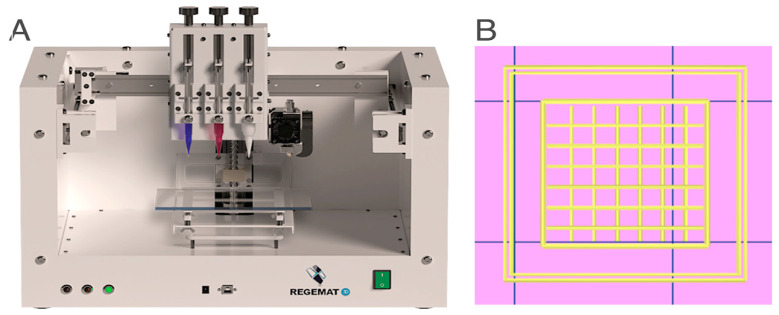
(**A**) Configuration of the Regemat 3D Bioprinter and (**B**) CAD model designed on the Regemat 3D software to print all the scaffolds.

To print the scaffolds an extrusion-based method featured in a 3D bioprinter (Regemat 3D BIO V1, Spain) (Figure 8) was used. Each produced hydrogel was loaded into 5.0 mL syringes with two different nozzle tips (0.58 mm and 0.41 mm diameter) and attached on the 3D bioprinter (Regemat 3D BIO V1, Spain). In addition, a design software (Regemat 3D Designer, Spain) was used to create the CAD model, to print layer-by-layer cube shaped scaffolds (width: 10 mm; length: 10 mm; height: 1 mm) with square pores (1 × 1 mm), a printing speed of 20 mm/s and a diagonal infill pattern with 90°.

To optimize the scaffold structure several parameters were changed, including the nozzle tip diameter (0.58 mm and 0.41 mm), the flow speed (2.5, 0.5, 0.25 and 0.15 mm/s) and the layer height (0.41, 0.25 and 0.15 mm).

### 3.4. Design of Experiments

In order to determine what percentage of starch would be the most suitable to be added to a 2.5 wt% solution of chitosan, a design of experiment tool was used. It consisted of a full-factorial design with two levels and three factors (percentage of starch, flow speed and layer height), 2^3^, three centerpoints (3). A total of 11 experiments were conducted (2^3^ + 3). For each independent variable, two different levels were studied, −1 and 1, where −1 corresponds to the lowest value used and 1 corresponds to the highest value used for each of the factors under study. To represent the centerpoint values, the numerical number 0 was used, which corresponds to the medium value for each independent variable. The design of experiments was produced and analyzed using an online software, desice.io (DoE in the cloud). Table 4 summarizes the levels and respective values used for each independent variable for the design of experiments and Table 5 summarizes the levels applied for each run.

For each of the experiments obtained, two different responses/dependent variables were analyzed, the total number of pores formed, designated as *NP* and the printability, designated as *Pr*. The total number of pores was obtained by counting the pores formed in each scaffold. The printability (*Pr*) variable is obtained by applying the Equation (1).

(1) $$Pr=\frac{L^{2}}{16A}$$

where *L* represents the perimeter of the pore in mm and *A* the area of the pore in mm^2^ [25]. According to this formula, the desired value for each scaffold is *Pr* = 1, corresponding to a scaffold with pores of good quality, good resolution, fidelity, uniformity and with well-defined boundaries [25].

To evaluate the quality and the conditions required to determine the responses for each experiment, the scaffolds obtained were photographed with a digital microscope (RoHS USB, Shenzhen, China). Since it is necessary to assess the area and perimeter of the pores formed, a calibrator with an accurate length of 20 mm was placed side by side with each of the scaffolds produced in further analysis through the image J software (version-1.53t; Wayne Rasband, Bethesda, MD, USA). Thus, the area and perimeter of the pores produced were evaluated using the pictures obtained (pixels) compared with the calibrator as the reference (mm).

The two responses used by the design expert presented some limitations related to the fidelity of the results, being necessary to find a new formula that allowed to relate and simultaneously evaluate the number of pores formed with their quality. Thus, a new response designated as “Quality of the Scaffold” (*Qscaffold*) was obtained through the deduction of Equations (2) and (3):

(2) $$Qscaffold=\frac{PrPr\;x\;NP_{scaffold}\;}{NP_{maximum}},\;\mathrm{if}\;\mathrm{Pr}\;\leq \;1$$

(3) $$Qscaffold=\frac{\left[1-\left(Pr-1\right)\right]\;x\;NP_{scaffold}}{NP_{maximum}},\;\mathrm{if}\;\mathrm{Pr}\;>\;1$$

where *Pr* is designated as the printability of the pores, *NP_scaffold_* is the total number of pores produced in the scaffold and the *NP_maximum_* is the theoretical total number of pores that could be produced in a scaffold, according with the geometric properties of the CAD model. For the present study, the *NP_maximum_* is 49 pores. A *Qscaffold* of 1 is the desired value and is the maximum possible value obtainable. Thus, a *Qscaffold* = 1 represents a scaffold with good appearance, strong structure, uniformity and with well-defined pores.

### 3.5. Viscosity Evaluation

To evaluate the viscosity of the pure chitosan hydrogels and chitosan–starch hydrogels, a laboratory DVE rotational viscometer (Brookfield Ametek^®^, DVEELVTJ) was used and in accordance with the profile of the hydrogel, a suitable spindle was chosen (Spindle number 4 S64). All the hydrogels were transferred to an adequate container suitable for the viscometer. All readings were performed in an incubator with controlled temperature at 25 °C by increasing and then decreasing the rotational speed value. To study the rheological profile of each hydrogel, all the measurements were analyzed by the construction of the respective viscosity (Pa.s) vs. shear rate (s^−1^) plot.

## 4. Conclusions

This work aimed at the formulation and production of chitosan-based hydrogels suitable to produce scaffolds with an extrusion-based 3D bioprinter. Initially, two hydrogels were produced only with chitosan, at different percentages (2 and 2.5 wt%). The hydrogel with 2.5 wt% showed better results compared to the 2 wt%. Although it was possible to print scaffolds with only chitosan, the final printability needed to be improved. Subsequently, new hydrogels were produced by adding different starch amounts. The design of experiments proved to be a very useful approach in the development of suitable chitosan hydrogels for 3D printing. The scaffolds obtained using a composite hydrogel (chitosan–starch) were shown to have a suitable structure, with well-defined pores that can be used to incorporate a desired active component in the matrix or loaded into the pores.

## Figures and Tables

**Table 1:** Design of experiments: number of pores and printability obtained for each experiment performed.

Experiment	Number of Pores (*NP*)	Printability (*Pr*)
**1**	34	0.82
**2**	15	0.74
**3**	10	0.84
**4**	0	0.00
**5**	0	0.00
**6**	1	0.89
**7**	6	0.94
**8**	19	0.82
**9**	24	0.82
**10**	32	0.93
**11**	12	0.82

**Table 2:** Design of experiments: Qscaffold values for each experiment.

Experiment	*Q_scaffold_*
**1**	0.57
**2**	0.23
**3**	0.17
**4**	0.00
**5**	0.00
**6**	0.02
**7**	0.12
**8**	0.32
**9**	0.40
**10**	0.61
**11**	0.20

**Table 3:** Number of pores, printability and Qscaffold obtained using chitosan 2.5 wt% and starch 13 wt% with a layer height of 0.41 mm, applying different flow speed values.

Flow Speed [mm/s]	Number of Pores	Printability	Qscaffold
0.25	40	1.05	0.78
0.15	46	0.99	0.93

**Table 4:** Levels and respective values used for each independent variable of the design of experiments.

Independent Variables/Factors	Levels		
	−1	0	1
Starch [wt%]	2.5	6.3	10
Flow speed [mm s^−1^]	0.15	0.2	0.25
Layer height [mm]	0.25	0.33	0.41

**Table 5:** Design of experiments; levels applied for each run.

Experiments	Starch [%]	Flow Speed [mm/s]	Layer Height [mm]
1	1	1	1
2	−1	1	−1
3	0	0	0
4	1	−1	−1
5	0	0	0
6	−1	−1	1
7	−1	−1	−1
8	0	0	0
9	1	1	−1
10	1	−1	1
11	−1	1	1

## Data Availability

Not applicable.
